# Selections and Abstracts

**Published:** 1898-08

**Authors:** 


### SELECTIONS AND ABSTRACTS.

---

### Should all Milk Used for Infant Feeding be Heated for the Purpose of Killing Germs? If so, at What Temperature and How Long Continued?*

Although in this paper I will present no new facts, I hope to be able to make my remarks interesting by presenting to you a summary of the answers I have received to the questions I sent to the members of the Society relating to the heating of milk for the destruction of germs. I wish also to express my thanks to the members of the Society for their very courteous co-operation, which has made it possible to present this summary of the opinions of many of the most prominent pediatrists of the country. The questions I sent out were rather hurriedly drawn up and were not perhaps as definite in their meaning as they should have been; but in general the answers received gave evidence that they were understood as was intended.

I have received 37 answers, of which 34 were categorical and 3 in the form of letters. One of these letters gave details of a process used by the writer in which the milk was twice boiled, while two letters gave no details, and were thus not suitable for this purpose.

In answer to the first question, "Do you consider that milk is rendered more digestible by sterilization or pasteurization?" the answers were: Yes, 3; a qualified yes, 1; no, 19; a qualified no, 4; while three thought it less digestible when sterilized, and four more digestible when pasteurized. The great majority, then, consider that heating does not render the milk more digestible.

In answer to the second question, "Is sterilization or pasteurization to be advised for this purpose?" the answers are as follows: Yes, 1; qualified yes, 1; no, 21; qualified no, 1; pasteurization, 5.

The third question, "Would you recommend that milk should

*Read by Rowland Godfrey Freeman, M.D., New York, before the American Pediatric Society, Cincinnati, June 2, 1898.

always be heated for this purpose?" was answered as follows: Yes, 3; qualified yes, 4; no, 25; qualified no, 1. It is thus evident that a very large proportion of these gentlemen believe that milk may be fed raw under certain conditions.

The fourth question, "If not always, when, if ever, is it imperative?" The answers may in general be grouped as follows: Always, 1; when not kept cold or in summer, 17; in gastric or enteric disorders, 4; when dairy hygiene is questionable, 12; during epidemic of scarlet fever, measles, cholera, or typhoid fever, 1; when milk is old, 3; in cities, 7.

The fifth question, "Do you prefer pasteurization or sterilization?" brought the following answers: Pasteurization, 23; pasteurization under certain circumstances, 6; sterilization, 1. Including the letter mentioned before, we would have 2 preferring sterilization; one prefers pasteurization in private practice and sterilization in hospital work.

As to the temperature for pasteurization: 1 uses 140° for 15 minutes; 1, 150° for 30 minutes; 2, 155° for 30 minutes; 1, 155°–158° for 15 minutes; 3, 155°–163° for 15–30 minutes; 1, 160° for 20 minutes; 1, 160°–167° for 20–30 minutes; 1, 160°–170° for 35 minutes; 1, 160°–177° for 20–30 minutes; 1, 160°–170 for 35 minutes; 1, 165° for 35 minutes; 18, 167° for intervals ranging from 6 to 35 minutes, the majority being from 20 to 30 minutes; 1, according to Walker-Gordon method.

The question concerning the duration of sterilization was answered by only 13 members, the time varying from 15 minutes to 1½ hours.

The last question, "Are there any practical disadvantages in heating milk for sterilization?" brought forth a variety of answers, as follows: Yes, 2; no, 2; less digestible, 7; sterilized milk less digestible, 3; less nourishing, 6; possibly a contributing factor in scurvy, 8; objectionable taste, 4; objectionable odor, 1; change in color of milk, 1; destroys emulsion, 1; cream not evenly mixed, 1; constipating, 2; household sterilization unsatisfactory, 1.

These replies seem to show a remakable unanimity of opinion of the members of the Society throughout the country, the predominating opinion being that raw milk would be the best food were it possible to obtain it clean, while a considerable number are evidently willing to take their chances with raw milk during certain seasons of the year and under certain conditions of dairy hygiene.

It was therefore surprising to me that more of the members had not made use of pasteurization at a temperature lower than 167° F. There seems ample evidence that 155° F. for 30 minutes (a temperature exposure which does not change the taste of the milk) is sufficient, but only six of the replies advocate this temperature. One answer, which coincides very nearly with my views, comes from Dr. Victor C. Vaughn, of the University of Michigan, whose authority in such matters is widely recognized. This I will take the liberty to present in full:

"DEAR SIR—I will answer your questions as follows:

"I do not think that milk is rendered more digestible by sterilization or pasteurization.

"2. Sterilization or pasteurization is not advised for the purpose of rendering milk more digestible.

"3. If milk could be obtained from healthy animal under complete aseptic precautions, I do not think it would be necessary or desirable to have it heated before feeding it to children.

"4. Practically, sterilization or pasteurization is imperative, because milk is not obtained at all times from healthy cows, and very rarely, if ever, under aseptic precautions.

"5. I prefer pasteurization to sterilization. Pasteurization should be carried out at a temperature of 155° to 158° F. When milk is heated to 160° F., it is so changed that a marked difference in taste is produced.

"6. I think that a temperature of 155° to 158° F., maintained for fifteen minutes, is sufficiently active to kill toxicogenic germs that may be present, provided that the milk, after having been heated, be kept at a very low temperature. The keeping of milk at a low temperature after heating and before it is fed to the child is, I think, absolutely necessary, because we know that even boiling does not destroy the spores of certain harmful germs in milk; but these spores do not develop at a low temperature, and there is reason for believing that these germs do not develop in the body.

"7. If milk is sterilized, I think fifteen minutes long enough time.

"8. There are practical disadvantages in heating milk for sterilization. Some of these practical disadvantages are inherent and others are accidental and avoidable."

It does not seem to me that our dairy hygine, even under the best circumstances, has reached a point where it can produce a raw milk which is absolutely safe food. Cow's milk must be obtained by pressure on the teats of a cow, and these teats hang beneath an udder which is covered with hair, and from the belly of the cow which is also cevered with the same hair-covered hide. Moreover, this portion of the cow is particularly liable to be soiled with dirt, as it comes in contact with the ground when the cow lies down. Its hairy covering, moreover, holds the dirt, which is gradually shaken out by friction. If the cow has loose fecal movements, these run down the inner surface of the thighs and the posterior portion of the udder. The contamination dries on the udder in the air, and during milking is apt to fall as dust into the pail. Moreover, the milk ducts of the cow may contain many bacteria, although usually contamination from this source is not great. In some cases, however, it is considerable, and then it is not always eliminated by throwing away the milk from the first few squeezes of the teat. I have found it present in the milk forced out by even the fortieth squeeze. Milk may thus become contaminated in the milk ducts, and at any rate has to be obtained from a bad immediate environment. Many efforts are being made to minimize these dangers, but with the best methods now used they still exist to a considerable extent.

But this is not all. The milkman's hands are almost never clean, and of necessity hardly can be. The milkman must labor hard with his hands all day, causing a thick, rough callus, which is difficult to clean, if an effort to clean them is made. His hands are employed in handling manure, and in attending to many duties involving contamination. Occasionally they are used during the day in waiting on some one sick with a contagious disease, and when such is the case the consumers of the milk are apt to suffer.

If wet milking is used, the milkman's hands are practically washed over the milk-pail.

We have here again in the mikman a danger to the milk which cannot yet be eliminated, and even with great care on the part of the dairy superintendent, it will be difficult to entirely do away with these dangers.

Epidemics due to milk have originated from a mild unrecognized case of typhoid in a milkman, also from a beginning diphtheria in a milkman. Such sources of danger may exist in very carefully conducted dairies, although the liability to them is much diminished.

It has seemed to me worth while to go over these dangers to milk and to consider the difficulty in eliminating them in connection with the matter of heating milk, to show how difficult this problem of obtaining clean milk really is.

The original contamination of milk up to the time of bottling in well-conducted dairies where great efforts are made to obtain clean milk, rarely amounts to less than five thousand bacteria in each C. C. By the time such milk reaches the consumer the contamination is still greater, and if, as is usual, the milk is used during the twenty-four hours following delivery, it is apt to be very considerable before a fresh supply arrives, and is probably, as a rule, something between fifty thousand and five millions a C. C., or is, roughly, between three thousand and three hundred thousand a drop. These bacteria are for the most part air bacteria, but they may be putrefactive bacteria, or toxin-producing bacteria, or pathogenic bacteria, and thus may produce in the infant that is fed on the milk a gastro-enteritis or acute poisoning, or the infectious disease of which the special organism present is the cause. Many instances representing each of these classes of illness due to milk have been reported.

Does it seem right, in view of what we know of the bacteriology of mother's milk, to give such contaminated milk raw to infants?

It has seemed to me that although fresh uncooked milk is the ideal and rational form for infant feeding, the practical impossibility of obtaining cow's milk clean has rendered some form of sterilization necessary. It does not seem fair to put into an infant's stomach a food containing thousands of bacteria in each drop, these bacteria being of unknown quality and very possibly of dangerous and pathogenic nature.

For the present, then, some sort of sterilization, it seems to me, must be used. High temperature sterilization causes certain chemical changes in milk. The change in the taste of milk occurs at 70° C. (158° F.), and the changes found by chemists begin with a temperature of about 80° C. (176° F.) Clinically, it has been observed that children fed on milk heated to a boiling temperature do not thrive as those fed on raw milk, and also that this bottled food seems to be a predisposing cause of scurvy. On this account, then, it would seem that the lowest temperature which is efficient should be used for sterilizing. Moreover, a lower temperature continued for a long time is as sufficient in its bactericidal action as a much higher temperature for a very short period. We should, then, use a temperature for heating which is the lowest which, when continued for a considerable time, will destroy with certainty all those pathogenic bacteria most feared in milk, as well as the bulk of the bacteria present.

As I stated before this Society two years ago, I believe that on the consideration just mentioned, 68° C. (155° F.) for thirty minutes, followed by a rapid cooling, is the best temperature exposure. Such a temperature will destroy the germs of diphtheria, typhoid fever, and tuberculosis, and so many of the other germs present that a plate planted from milk so treated and kept at a laboratory temperature will usually show no growth in twenty-four hours. At the same time this milk has not been heated sufficiently to give it a "cooked-milk" taste or to change its taste at all, and the temperature to which it has been exposed is more than ten degrees centigrade below that at which the chemical changes in milk due to heating are said to take place. It is, however, evident that pasteurization at this temperature has been as yet but little used. I would urge on those gentlemen who have used a temperature of about 167° F., and who are inclined to favor raw milk, to try first pasteurization at this lower temperature. In using this temperature care must be exercised that, approximately, this temperature is sustained for half an hour, and equally that the milk is immediately and rapidly cooled and kept cool. Raw milk, I believe, cannot yet be considered a safe food.—*Pediatrics.*

---

### PROFESSOR SCHENCK'S RESEARCHES ON THE PREDETERMINATION OF SEX.

The pamphlet opens with the statement that it is impossible to command natural processes, but possible by scientific means to exercise a more or less effectual influence upon them, in order to extract from them the best possible results. His essay falls into three parts—a summary of the writings of his predecessors, an account of his own researches and deductions, and finally a description of the method of treatment he has devised, with illustrative cases.

In the development of an embryo the generative organs are at first indifferent—hermaphrodite; in the further process of growth one set develops while the other atrophies. This tendency must be predetermined from the time of fertilization, for each cell formed from the ovum must have sexual characters, since these are not confined to the generative organs, but appertain to the whole body. The readiness with which an ovum can be fertilized depends upon its position in the ovary, the thickness of its envelope, etc., and these may also have a bearing on the question of the sex. In other words, the predetermination may precede fertilization, and of this confirmation is found in the development of bees and in the production of male and female flowers by plants under different nutritive conditions. In this connection Professor Schenck enunciates and discusses at considerable length the views of previous writers. He points out that the male sex preponderate to a definite though slight degree in the total number of births, and that the sex of the child is more likely to be that of the older parent. He pays particular attention to the theory of crossed sexual heredity, by which each sex tends to propagate the other. Thus, if the sexual power of the male be greater, a female offspring is more likely to result, and *vice versa.* This theory is threshed out most thoroughly and with abundance of quotations and examples. In the end Professor Schenck practically accepts it, and makes use of it in his further work.

With regard to the influence of environment upon sex, he quotes Robin's statement that in warm climates females predominate, in cold and uncomfortable, male. Born also showed that ninety-five per cent. of artificially fertilized frogs' eggs hatched out as females, this being an effect of nutritive conditions acting after fertilization. Thury's researches are fully analyzed, and are stated to have originally called Professor Schenck's attention to the subject. Thury found that cattle fertilized at the beginning of "heat" threw more females; at the end, more males. This he explained by the degree of ripeness of the ovum, but Schenck accounts for it on the crossed-inheritance theory, the sexual power of the female being at its greatest at the end of the period of rut. This part of the work is summed up in the statement that the sex of offspring largely depends upon the state of nutrition of the parents, particularly that of the mother during pregnancy. During this period the difference between intake and excretion represents the food of the embryo, and hence requires special attention. The temperature is slightly raised owing to the oxidation processes, which entail a considerable consumption of red-blood corpuscles, and consequent diminution of hemoglobin.

The second section begins with the enumeration of the fact observed in domestic animals and in insects, that the better the mother is nourished the more females she produces, the number of males remaining practically constant. This influence upon the fetus in utero has received but little attention from the practical point of view, and Schenck consequently set out upon a series of observations based upon the theory of crossed sexual inheritance. He first investigated the excreta, and particularly the carbohydrates of the urine. The presence of a certain amount of sugar, which is commonly recognizable by the phenyl hydrazine test in perfectly normal individuals, indicates incompleteness of the oxidation processes, whereby a certain quantity of heat is lost to the body. This physiological output of carbohydrates is in the male sex most marked during the period of growth—that is, between the ages of fourteen and nineteen. In women there is no corresponding increase, but small quantities may appear in the urine before and after menstruation, while Iwanoff and others have shown that glycosuria is common in pregnant and parturient women. Now the amount of sugar normally excreted is equal in men and women, but more significant in the latter, owing to the lesser activity of their metabolic processes. For the perfect ripening of the ovum it is necessary that oxidation shall be perfect—that is, that no sugar shall be left unburnt. Where there is a remainder of unburnt sugar the ovum stands a chance of being less ripe and less well nourished. Hence the properties of its protoplasm are less well developed, and by the theory of crossed inheritance it is more likely to produce a female child. On the other hand, when the urine is free from sugar the ovum can attain perfect development and give rise to male offspring. It is upon this cardinal principle that Schenck's theory is based. He holds that a prolonged course of appropriate nourishment, both before and after fertilization, will tend to the conception of male children only.

The next question is of the means to be adopted to insure this end. If a male child is desired, and the maternal urine contains no sugar, but abundance of reducing substance (particularly the levorotatory glycuronic acid), he allows impregnation forthwith. If, on the other hand, sugar is present, it must be removed, and the reducing substances increased, before fecundation can take place. It is found that the urine of a woman pregnant with a boy contains more reducing substances than that of one with a girl. We need not enter into details of the diet recommended, beyond saying that it contains a large amount of proteid, which seems to be required by a male embryo.

Finally, Schenck gives what may be called his clinical results. He quotes numerous cases to show that the bearing of female children is associated with glycosuria. In such instances he recommends a diet comprising plenty of proteid and fat, and as little carbohydrate as can be tolerated; this must be taken for two or three months before and three months after impregnation. He gives an example in which six boys were born in succession under this treatment, and a girl immediately it was relaxed; and others in which boys were born after repeated births of girls before the treatment. In all, out of seven recorded cases six were successful. He concludes that the nutrition of the mother plays a most important part in the determination of sex, and that in countries where much flesh is consumed there is a marked preponderance of male children. This can be imitated artificially, but it is far more important to insure the completeness of oxidation processes in the body. As long as the combustion of the food is perfect and the urine is totally free from sugar, the exact amount of meat consumed is of secondary importance. The birth of male children can thus, in certain cases, be predetermined, but the voluntary production of girls is a problem yet unsolved.—*British Medical Journal.*—*Medical Review.*

---

### INHALATION OF VINEGAR TO CONTROL NAUSEA AND VOMITING AFTER ANESTHESIA.

Many and varied are the methods proposed and used to overcome the nausea following the administration of an anesthetic, but one of the simplest, and in my own experience one of the most satisfactory, methods of controlling this condition has been the administration of strong vinegar by inhalation. The use of vinegar in this manner for vomiting was first proposed in 1829, and was practiced from time to time by various surgeons, but it remained for Mackenrodt to apply it extensively, he probably having adopted it from the recommendations of earlier surgeons, who lived in both the pre- and post-anesthetic days. Its beneficent action is explained by Lewin as due to the neutralization of the free chlorin, one of the products of chloroform, by the acetic acid. The chlorin acts as a marked irritant to the pharyngeal mucous membrane and induces vomiting, but it is neutralized by the acid, which soothes the irritated parts as well. Ether, however, is much more directly irritating to the respiratory passages during inhalation, but the vinegar gives as satisfactory results after it as after chloroform narcosis. The simplest explanation of its good effects is that its pungency stimulates—it being too dilute to exert any irritative action—the respiratory mucous membrane and promotes the normal secretions, and, by its soothing action upon the peripheral nerves of the parts, lessens the irritability of the pneumogastric or its centers, and the reflex condition of vomiting is controlled. Furthermore, that vinegar is a restorative and soothing stimulant to the respiratory tract and to the nervous system, is well attested by its widespread use among the ladies in their vinaigrettes in place of "smelling salts." In certain countries the pungent qualities of the aromatic vinegar are used almost to the exclusion of the ammonia or lavender salts, and all because of the more refreshing effects following its use.

Whatever the correct explanation may be, certain it is that, in cases which have been properly prepared for operation, and whose stomach has not been filled with blood during the operation, it almost, if not completely, prevents vomiting. The method of administration is by saturating a towel or cloth with fresh, strong vinegar (preferably that made from cider), and holding it a few inches above the patient's face, or hanging it from the bedstead so that it will be near his head. It should be used directly after the anesthetic has been discontinued, and kept up continuously for hours.

In one case, to which ether had been given, nausea began soon, but ceased in about one and one-half minutes after using the vinegar. This was then removed and the nausea returned, but again disappeared after the vinegar was given. The action was so marked that the process was repeated five or six times, so as to verify the conclusions, and each time the result was the same as first noted, the patient quickly becoming quiet as though going under complete anethesia.

Another case was given chloroform for the removal of pharyngeal growths, and swallowed considerable blood. Vomiting of the clotted blood occurred, but ceased immediately after and did not return.

These have been duplicated by about twenty-five or so others in whom the action was almost uniformly beneficial. The relief from thirst to the patient is most marked, and the refreshing effect is both grateful and welcome to the sufferer. Its simplicity and efficiency commend its use to all having aught to do with such cases. It is also free from any toxic effects, and can occasion no harmful conditions.

### THE COMPRESSION TREATMENT OF PULMONARY TUBERCULOSIS.

The recent address of Dr. Murphy, of Chicago, before the section in surgery of the American Medical Association was a most notable contribution to the subject of pulmonary surgery. The address was read only in abstract, and it is to but one feature of it that we are now going to call attention, namely, the part relating to the treatment of tuberculous disease of the lung by compression of the organ. As we understand the matter, Dr. Murphy proceeds upon the assumption that a tuberculous lesion of the lung, like one of a joint, for example, may ordinarily be healed quite readily by securing immobility, functional rest, of the affected part. In pursuance of this idea he immobilizes the lung by compressing it, crowding it back upon its hilum, establishing a sort of artificial atelectasis. This he accomplishes by injecting a quantity of nitrogen into the pleural sac. Nitrogen, he finds, neither exerts any untoward effect upon the pleura nor is absorbed to any appreciable extent; it simply keeps the pleura distended and the pulmonary tissue compressed. It is said that during the continuance of the compression the patient feels remarkably free from the symptoms that had previously preyed upon him.

The gas is allowed to remain in the pleura for a period of several weeks, and then it is withdrawn. In a goodly number of instances the symptoms do not return, and the inference is drawn that the disease has been overcome. The lung again becomes aerated and expands almost if not quite to its normal size. If, on the removal of the nitrogen, the morbid symptoms return, more of the gas is thrown into the pleura and kept imprisoned there for another term of weeks. It is said that this second injection is by no means always found necessary, and that when it is called for it almost invariably suffices for the cure of the disease in that lung. Then the other lung is treated in the same way.

We have here given only a general outline of Dr. Murphy's plan of treatment. For the details, upon which much undoubtedly depends, the profession will have to wait until the address is published. One may picture to himself a number of difficulties in the way of success with this method of treatment, but not one of them seems to be insurmountable. The first that occurs to us is the task of passing the eye of a hypodermic needle through the parietal pleura without at the same time passing it through the visceral layer, provided there is no pleural effusion. It is pointed out, however, that the outer layer of the pleura is held firmly attached to the wall of the chest, while the pulmonary layer is free to yield before the pressure of a blunt-pointed needle, and so escape puncture. Still, we may suppose that adhesions in the immediate neighborhood of the puncture might seriously impair this evasive yielding of the pulmonary pleura. Nevertheless, whatever difficulties might be encountered in the attempt to make the eye of the needle pass the parietal pleura and stop short of the visceral in the ordinary way of inserting the instrument, they might all be overcome readily, we presume, by cutting down methodically and picking up the outer layer of the serous membrane with a forceps.

Another difficulty that has been suggested lies in the frequent presence of extensive pleuritic adhesions, so extensive as practically to abolish the potential cavity of the pleura. But this state of things, we understand, Dr. Murphy meets by breaking up the adhesions—a procedure which is no doubt thoroughly practicable in a great many instances. We have heard this further objection mentioned, that the preponderance of tuberculous lesions in the apex would render the majority of cases refractory. This, we presume, rests on the assumption that the compression exerted by the nitrogen would be subject to the limitations of that exercised by plural effusion—an assumption that seems to us not wholly warranted, At all events, when so careful a man as Dr. Murphy has been experimenting with the treatment for three years, as we understand he has, and when he reports a number of cases of cure, all such theoretical objections as those we have mentioned must be held in abeyance until the distinguished author's full data have been laid before us and until the plan has had ample trial in his hands and those of others. The present time seems to be an era of glorious possibilities in the treatment of tuberculous disease, and more than one of them, we think, will be realized. The chances are, it seems to us, that Dr. Murphy's ingenious plan will turn out to be one of those from which decided advantage may be derived.—*N. Y. Medical Journal.*

### Surgeon Nicholas Senn.

"Lieutenant-Colonel Nicholas Senn, U. S. Volunteers, chief surgeon Sixth Army Corps, under command of Major-General Wilson, Camp Thomas, Chickamauga," severs his connection with the National Guard of Illinois and announces the same to the medical profession at the close of a six-page letter published in the *Association Journal* of June 11th.

It is a remarkable incident of our volunteer service, the most eminent of American surgeons—an author and teacher of repute, a man of wealth acquired in his profession, the donator of vast medical libraries to the public library of his adopted city—enlisting in the volunteer service of his country. And yet it seems easy, natural and appropriate.

Senn has sucked the Chicago orange dry; he has reached the top round of the ladder of surgical fame. He is without opposition; his social and medical status is assured; he has legions of students, readers and admirers; he was president of the American Association at its greatest celebration, and in his address stood for the old code without changes, and did all that his commanding personality could accomplish to insure the perpetual autonomy of the county and state organizations which have their annual expression in the American Medical Association, the mightiest and most influential of medical gatherings among English-speaking people. What is it to Senn, Western autochthon of the surgical world, that he gives another winter of clinics and lectures in the arenas of Rush or Cook County Hospital, or compiles his annual treatise, or takes another winter month off to go hunting again in Texas?

But war, always alluring to men of genius, discovering to the nations such as Grant and Lincoln, has attractions and possibilities even for those who have accomplished great achievements in civic life. The greater the man the greater the opportunity. The best are none too good for the uses of patriotism. Senn is fond of travel and of history; witness his observations of Pompeiian surgical instruments, of the Græco-Turkish war. Here, indeed, is a great opportunity—to carry aseptic surgery into camp and field; to employ the modern prophylaxis against infectious disease; to direct the largest possible surgical and medical clinics since the civil war; to measure the medical and surgical skill of our army medical corps against the magnificent accomplishments of the Germans in the Franco-Prussian war, and also to see what may be done to protect great bodies of troops from temperate zones against the plagues of tropical cities, coasts and climates.

Finally, there is the joy and glory of being part of the actual thing, of chronicling the results and employing them in after work, like Æneas in the Trojan war, to be able to say, "All of which I saw and part of which I was." There is no more romantic figure among our surgeons of the civil war than that of McFadden Gaston, who treated the first Union soldier injured when Anderson saluted the American flag as it was taken down for four years at Fort Sumter, who was loyal to his Southland through the civil war, and whose last military service—such are the accidents of civil war—was caring for United States soldiers and sailors on shipboard between New Orleans and New York after the surrender at Appomattox. What a richness of story and honorable reminiscence!

But to go with certainly conquering troops, with the approval of the nation, and, in the observation of the medical and civic world, as the acknowledged surgeon of the decade, rich in every knowledge and expedient, and with the medical and surgical resources of the greatest nation in history at one's beck and call—why not?

Who would stay at home when every opportunity is offered, when there is no personal sacrifice, and when there is infinite necessity and possibility of alleviating pain and saving life, and at the same time of laying up material and experience for the future? No surgeon is too great or too useful for his country's good.

Surgeon Senn's letter to the *Association Journal* comprises, first, a dissertation on war and an enthusiastic eulogium on the justice and conduct of the Spanish-American war; second, a description of Camp Tanner, at Springfield, and a glowing tribute to Governor Tanner and his wife—the Governor "a worthy successor to Yates and Oglesby"; then, third, a description of the examinations of the seven volunteer regiments, and a list of the examination questions in the medical department. Ten thousand men were examined at the rate of nine hundred daily. The common causes for rejection were hernia, varix, poor physique, heart disease, lack of chest space, loss of teeth, and flat-foot. Varicocele was present in twenty-five per cent. of the cases, but only two were rejected on this account. Flat-foot was very common, but few were rejected, as, like the varicocele patients, they were not impaired by the deformity. The rejections for all causes were less than ten per cent., and more were rejected from the country troops than from the city. Frequently the rejected "were pale, speechless and staggering on learning that they were deprived the great boon of defending the honor of their country."

The medical staff were put through a school of instruction from 8 to 10 daily, with operations on the cadaver. A score of lectures were given on proper topics—sterilization, sunstroke, blood-stopping, dysentery, anesthetics, shock, prevention of camp-diarrhea, etc. The vaccination rules are admirable, and prevented septic complications. Three cases of cerebro-spinal meningitis, with death, are reported on at length; it was sporadic and controllable.

The pneumonia present was malignant; two out of thirty-two cases died. Measles and mumps were controlled by prompt isolation. Assistant Surgeon Cole, of the Sixth Infantry, died at Fort Wayne, Ind., of pneumonia, while en route to Washington.

The entire report is of the highest interest; it is sane, fervid, scientific and enthusiastic. Senn will have an enormous influence in the volunteer service upon the medical staff. His presence will inspire confidence and give tone to the medical and surgical work. Plain of speech, modest in manner, genial and approachable, known throughout the medical world, and without personal motive, actuated only by scientific, humanitarian and patriotic motives, he will be a commanding figure in the medical and surgical history of the present war.—*Indiana Medical Journal.*

---

### NORMAL SALT-SOLUTION—THE VARIOUS METHODS AND INDICATIONS FOR ITS EMPLOYMENT.

Dr. P. Findley concludes an article on the above subject with the following summary (*Med. Stand.*):

1. When normal salt-solution is indicated, enteroclysis is the method of choice, providing there is time to await its effect.

2. The body-temperature, vascular tension, renal, cutaneous, and intestinal secretions are influenced in direct ratio to the temperature of the injected fluid.

3. Injected solutions of high temperature, however, may lower the body-heat by promoting the excretions.

4. A solution of 60° to 70° F., given within the colon, will first stimulate and later depress the blood-tension and the secretions of the skin and kidneys. It is therefore to be used with caution, particularly in renal insufficiency.

5. In the subcutaneous method we have all that is required, when immediate effect is desired, except when abdominal section may indicate intraperitoneal injection; where the withdrawal of a quantity of blood has made it possible to give intravenous injection with the least possible loss of time, and where the serous cavities have been relieved of a quantity of fluid which may be replaced by a normal salt-solution.

6. As a rule, no time is gained by the employment of the intravenous method, which should only be used when preceded by venesection for the withdrawal of a quantity of blood.

7. In intravenous injections it is possible to cause death from too great dilution of the blood—an accident quite impossible in hypodermoclysis or enteroclysis.

8. Normal salt-solution is indispensable in the treatment of alarming hemorrhage, and is of great value in the treatment of the various toxemias, and in renal insufficiency.

9. After the removal of a large quantity of fluid from the pleura cavity, the salt-solution may be injected into the cavity as a substitute for the effusion, and will thereby lessen shock and relieve septic infections.

10. In cholera and cholera infantum, normal salt-solution is invaluable as a substitute for the lost serum.

11. Venesection with the withdrawal of a quantity of toxic blood is indicated in toxemias, where the patient is plethoric, and should be followed by intravenous injections of an equal or greater amount of normal salt-solution.

12. In hemorrhage normal salt-solution maintains the circulation by adding to the volume of the circulating fluid, which would otherwise stagnate in the veins, because there is not sufficient volume for the heart to propel.

13. In toxemias normal salt-solution dilutes the toxins of the blood and favors their elimination by stimulating the excretory organs.—*Charlotte Med. Jour.*

---

### THE MANAGEMENT OF PATIENTS BEFORE AND AFTER LAPAROTOMY.

Wiggin (*Medical Record*) concluded his paper on this subject as follows, calling attention to the points which he considers important:

1. The importance, whenever practicable, of prolonged preparatory treatment of patients about to undergo an abdominal operation.

2. The importance of the administration of cathartics in the early part of this period, followed by large enemas for the purpose of cleansing the intestinal tract.

3. The importance of keeping a record of the bodily temperature, respirations and pulse-rate for several days in advance of the operation, and of making a final examination of the urine.

4. The necessity in the female of arranging to have the operation performed a few days after the menstrual period, and the cleansing of the vagina, even when it is intended that the operation shall be by the abdominal route only.

5. The administration of a small quantity of peptonized food (one ounce) containing stimulants two hours before giving the anesthetic, for the purpose of lessening the tendency to nausea and vomiting after the recovery of consciousness.

6. The necessity of the anesthetic being given by an experienced physician and in the smallest possible quantity.

7. The necessity of protecting the patient's body properly with clothing and blankets during the operation.

8. The advantage of stimulating the pulse before the heart has become much exhausted, and of using intravenous saline injections before the radial pulse has become extinct.

9. The leaving in the abdominal cavity, after a free irrigation, of a quantity of hot saline solution, for the purpose of stimulating the patient, preventing (?) the formation of intestinal adhesions, and lessening the danger of septic infection of the peritoneum.

10. The necessity of making the patient comfortable by change of position during the first two days of convalescence, and by the use of the rectal tube.

11. The necessity for early administration of food in reasonable quantities and at proper intervals.

12. The necessity of withholding stimulating enemata after operation in which extensive and firm pelvic adhesions have been broken up.

13. The necessity for deliberation as to the wisdom of reopening the peritoneal cavity in a given case of supposed concealed hemorrhage.

14. The importance of washing out the stomach as soon as the diagnosis of intestinal paresis is made, and of the persistent use of saline cathartics till the bowels move.

15. The importance of not administering cathartics to those convalescing from abdominal operations and who are pursuing a normal course too early or in too large doses.—*Charlotte Med. Jour.*

---

### Injections of Alcohol in Carcinoma.

A detailed description and careful estimate of the results thus far obtained from the treatment of cancer by injections of alcohol, is given by Sajous, in the *Monthly Cyclopædia of Practical Medicine* for January. Beginning with the eighteen cases of mammary carcinoma treated by Hasse twenty years ago—with fifteen complete cures and no recurrence—the survey closes with Kuhs recently reported cure of primary cancer of the nasal pharynx. The author decides that this last case, added to the others, "establishes alcohol on a basis seldom equaled by any agent proposed. It is safe to state that if tuberculin had had to its credit but half of the *bona fide* points already noted in favor of alcohol in the treatment of cancer, it could have withstood the test of time." The remedy acts by forming a consecutive-tissue capsule around each growth, causing obliteration of the blood-vessels and contraction of the neoplastic tissues. According to Hasse, the effect on the general health is even more surprising. The pain and uneasiness pass away, and sleep, appetite, assimilation and strength return in a most remarkable manner. By employing alcohol in different varieties of tumor, rapid reduction in the size and growth has been produced, but it was found that if too much be injected at one time, sloughing of the growth and general intoxication of the subject will follow. To secure a successful result, the treatment must be carefully conducted.

In the cases reported as cured by him, Hasse injected a mixture of thirty parts of absolute alcohol to seventy parts of water twice a week around the tumor, as well as into any infiltrated glands. The quantity injected varied according to the size of the neoplasm, and sometimes reached twenty Pravaz syringefuls. The only inconvenience observed was pain (for which local or even general anesthesia might be resorted to) and, occasionally, slight intoxication. In order to avoid making the injection into a blood-vessel, Hasse inserted the syringe-needle deep into the tissues, then unfastened it, leaving the canula in place. He then waited a moment; if the blood did not issue from the canula he readapted the syringe and made the injection; but, if blood did flow out, he removed the needle and made another puncture elsewhere. Under the influence of these injections the tumor diminished in size and soon became less painful. The treatment should be continued for some time after apparent cure, at intervals more and more prolonged.

In conclusion, an earnest plea is advanced that alcohol be given the faithful trial in this affection which it seems to merit.—*Medical Times.*

---

### THE NON-SURGICAL TREATMENT OF PYELITIS.

Dr. Robin writes that though pyelitis is usually and rightly considered a surgical affection, there are nevertheless many cases where medicinal measures may be employed with great benefit (*Bull. gén. de Thérap.*, April 30, 1897). The treatment may be divided into hygienic and medicinal. The hygienic treatment consists of an absolute milk diet, of light broths, of mild alkaline waters, and of stimulating the functions of the skin. For this purpose the author recommends vigorous rubbing of the skin with the following liniment :

| | |
|---|---|
| Tr. of cinchona } | aa 100 dr. |
| Spirit of camphor } | |
| Tincture of nux vomica | 25 dr. |
| Menthol | 1 dr. |

As a direct counterirritant over the region of the kidney hot irons are recommended, but cantharidal plaster is prescribed, as the possible absorption of the cantharidin may cause or aggravate any existing kidney trouble. Internally benzoic acid or sodium benzoate is administered in small dose (15 to 20 grn. in twenty-four hours), or balsam of tolu, copaiba or eucalyptol. As astringents are administered krameria, tannic or gallic acid, and opium. As an internal renal antiseptic salol is recommended.

For hematuria the following combination is recommended :

| | |
|---|---|
| Ergotin | 4 dr. |
| Oil of turpentine | 4 dr. |
| Gallic acid | 2 dr. |
| Simple elixir | 100 dr. |

Dose : One or two teaspoonfuls every 3 hours.

If the urine contains too much pus, large doses of quinine are to be taken. If the pain is too severe an opium suppository is to be used per rectum, and a liniment consisting of camphorated oil, chloroform, opium, hyoscyamus and belladonna is to be rubbed over the region of the kidney.—*American Medico-Surgical Bulletin.*

---

SERUM THERAPEUTICS AND PROPHYLAXIS OF YELLOW FEVER.

Sanarelli reports very encouraging results with his serum in an epidemic at S. Carlos do Pinhal. Twenty-two patients were treated with it, with six deaths; a mortality of 27 per cent., instead of the average 45, and this merely the first tests of the serum, determining doses, concentrations, etc., step by step, and administering it to several patients evidently beyond all help before being seen. In one case abrupt convalescence followed a single injection the second day. He considers the serum bactericidal rather than antitoxic in its effects, and obtained most striking results with intravenous injections of large doses. In three of the fatal cases the bacillus icteroides was isolated from the blood during the agonic period, by using the serum reaction method. He finds the horse best adapted to the production of the serum. He is especially encouraged by the results at the prison where yellow fever attacked a prisoner and two guards, all early fatal cases, but prompt prophylactic injections arrested the disease at once, and no further case occurred. He adds: "The instantaneous suppression of this focus of infection was obtained in spite of the most unfavorable conditions and the lack of active serum, the horse-serum being all exhausted and nothing left but the much weaker beef-serum. The persons who received the injections were also either mostly unacclimated strangers or in wretched physiologic conditions owing to the depressing and extremely unhygienic surroundings, and thus exceptionally predisposed to the disease." His report is published in full in *O Brazil Médico* of April 1 and 8. He vouches strongly for the absolute harmlessness of the prophylactic injections, from experience on himself as well as on others. His facilities were limited, as he only had a few animals at his disposal.—*Jour. Am. Med. Asso.*

---

### THE PHYSICIAN AS A BUSINESS MAN.

A medical journal of the present day is hardly complete without some reference to the physician as a business man, and when there is so much smoke there must be fire. The commencement day orator, who used to proclaim the physician's calling above the mere strife for "filthy lucre," now calls attention to the fact that the physician has been woefully negligent to himself and family by not combining business principles with his noble calling. A good deal of this poor management can be laid to the men old in practice—those who sent in their bills once a year or semi-annually. This plan may be well enough in rich farming communities, but in the larger towns and cities, if carried out, the doctor's cupboard will soon be like "Old Mother Hubbard's." The people in the larger cities, to quite an extent, "fold their tents like the Arabs and as silently steal away," cheating the landlord as well as the doctor. There is no reason why the physician should not send his bills monthly, and if no response is made during the following month go and find out where the trouble is. Short accounts make long friends, and the man who owes the doctor a bill is ever ready to damn the poor doctor with faint praise. The writer has collected at least eighty-five per cent. by following up accounts close, and people do not take offense so much as one might imagine. It is a good plan to carry in the pocket a small book containing a list of debtors, with amount due, and when some anxious (?) debtor meets you on the street there will be no need of telling him to come to the office so you can look it up. Never haggle over your bill, but state the amount and stick to it.—*Charlotte Med. Jour.*

---

### NARCOTIC NOTES.

The *Journal of Inebriety* (April) has the following:

Investigations in France go to show that the mortality among children of women working in tobacco is considerably more than double that of children of other working women.

Of 1,000 cases of the morphine habit—650 men and 350 women—the medical profession supplied forty per cent.

Morphinomania occurs most commonly between twenty-five and forty.

The cocaine slave is far more depraved than the alcoholic. He has no moral sense. He will lie for the mere pleasure of lying and will steal without purpose.

Patients should never know they are taking cocain. It should not be prescribed for asthma, hay-fever, etc., conditions in which it is at best merely palliative.

"Knock-out drops" are found to be uniformly highly concentrated solutions of chloral.

Bromides used for their sedative effects should be accompanied by hot baths and salines.

Chloral should not be combined with bromides for sedative effects upon inebriates.

Stop bromides with fall of temperature and feeble heart.—*Medical Age.*

### THE HYPODERMIC INJECTION OF QUININE.

Professor V. Stoffella, of Vienna (*Gazette hebdomadaire de médecine et de chirurgie,* May 22d), points out that the insolubility of quinine has for a long time prevented its use hypodermically. Some time ago an Italian physician discovered that quinine hydrochloride dissolved easily in association with antipyrine. This fact, which was confirmed by Gessard, was applied by Laveran, who prescribed the following formula for hypodermic injection:

| | | |
|---|---|---|
| ℞ | Quinine hydrochloride | 45 grains. |
| | Antipyrine | 30 grains. |
| | Distilled water | 1½ drachms. |

Professor Santerson, of Stockholm, showed that in reality a new combination was thus formed—viz., quinopyrine—whose toxicity was less than that of quinine. The author has, however, found the association of quinine with antipyrine useless and has employed hydrochloride of quinine alone for some years after the following plan: He puts thirty grains of hydrochloride of quinine, of whose alkaline reaction, and this he deems very important, he has previously satisfied himself, in a test-tube containing a hundred and fifty minims of distilled water. The test-tube is heated gently, and at a temperature of about 105° F. the solution is complete and is maintained for some time. On cooling, the quinine is precipitated in the form of a whitish mass, which redissolves with heat, or on plunging the test-tube into hot water. The injection is quite painlesss and besides, as the drug remains soluble at the blood temperature, there is not, as is the case with injections of quinopyrine, the induration at the point of injection. A Pravaz syringe contains under these conditions about three grains of hydrochloride of quinine, which may be given three times a day.—*N. Y. Med. Jour.*

---

### CARE OF PATIENTS AFTER THE OPERATION FOR APPENDICITIS.

J. M. Barton, M.D., *Philadelphia Polyclinic,* separates the cases into four groups · (1) where the abscess is opened without entering the peritoneal cavity; (2) where an operation is performed between attacks and no pus is present; (3) where general peritoneal cavity is opened and the abscess emptied ; (4) where the general septic peritonitis exists at the time of operation. In all operations for appendicitis there is but little danger from shock, and none from hemorrhage, after the operation is finished. If there is any shock it will readily yield to heat and strychnia. Death is caused by general septic peritonitis. There is some difference in treatment of each class, but speaking generally (not saying anything of the last group) the treatment consists of perfect rest in bed, no food at all for twenty-four hours, and but a limited amount of water. By the third day he can have ordinary diet in moderate amounts, such as soft-boiled eggs, stewed chicken or mutton, milk and dried toast, etc. The soiled dressings should be removed once or twice daily, but syringing out of the cavity is not advisable. The stitches may be removed from the seventh to the ninth day; at this time the drainage-tube (if one has been used) may be shortened and taken out the fifteenth day. There is no hurry about the bowels being opened, and under no circumstances is it advisable to purge for several days. To prevent hernia the wound should be strongly supported from the first by a rubber plaster fitted with tapes, and continued for months.—*Medical and Surgical Bulletin.*

---

### AMYLOLYTIC FERMENTS.

In an article on this important subject Wyatt Wingrave, M.R.C.S., Eng. (Assistant Surgeon to the Central London Throat and Ear Hospital), in the London *Lancet*, May 7, 1898, we are informed of a personal necessity that arose in the writer's experience for a reliable starch digestant. A crucial comparative examination was therefore made of many malt extracts and of Taka-Diastase, the tests being conducted both chemically and clinically.

He summarizes briefly: 1. That Taka-Diastase is the most powerful of the starch or diastatic ferments and the most reliable, since it is more rapid in its action—*i. e.*, "it will convert a larger amount (of starch) in a given time than will any other amylolytic ferment." 2. That Taka-Diastase seems to be less retarded in its digestive action by the presence of the organic acids (butyric, lactic, acetic), and also by tea, coffee and alcohol, than are saliva and the malt extracts. This is an important point in pyrosis. 3. That all mineral acids, hydrochloric, etc., quickly stop and permanently destroy all diastatic action if allowed sufficient time and if present in sufficient quantities. 4. That Taka-Diastase and malt diastase have, like ptyalin, no action upon cellulose (uncooked starch). All starch food should therefore be cooked, to permit of the starch ferment assisting nature in this function.

### MALARIA.

In a clinical lecture published in the *Tri-State Medical Journal and Practitioner,* Dr. Francis Delafield, of New York, says: "An arrangement which works here in New York is a combination with the quinine of acetanilid and arsenious acid:

℞ Quininæ sulphatis ........................................ gr. iij.
Aicidi arseniosi ........................................ gr. $\frac{1}{30}$.
Acetanilid ........................................ gr. ij.
M. et ft. in capsulum No. j.
Sig.: Take four in the twenty-four hours.

"Now, why this combination is better than quinine alone, or when arsenic is added, or why adding two grains of acetanilid makes it more efficacious than one-thirtieth of a grain of arsenious acid and three grains of quinine, is a question I cannot answer. I have found that it is so by personal experience; I found that the adding of the acetanilid gave me such results that I could not get along without it. I do not give large doses of quinine alone. I have been treating malarial fever in New York for twenty years, and I find that just such a combination of drugs answers very well in my hands; it is even better than Warburg's tincture."

### PREVENTION OF GLAUCOMA.

The etiology of glaucoma is still disputed, but Schoen, the new professor of ophthalmology at Leipsic, asserts (*Wien. Klin. Rundsch.,* Nos. 26 to 31, 1897; (*Am. Jour. of Surg. and Gyn.*) that no one is obliged to lose his sight from this cause. It can always be prevented if the eyes are seen in time by an expert and his warnings heeded, as the invariable cause is excessive strain in the effort of accommodation, which, of course, increases with age. The particulars of the last one hundred and forty cases he has treated are: Forty-eight per cent. hypermetropic; not one had possessed a distance lens. Astigmatism was present in 33 per cent.; in none had the astigmatism been corrected. In 20 per cent. there were no glasses, or they had been utterly inadequate. Nearly twice as many cases of glaucoma occur among women as in men, the former shrinking from wearing glasses until too late. While the excessive strain above mentioned produces anatomic changes which lead directly to glaucoma in time, yet any constitutional morbid tendency, any weakening or depressing cause, violent coughing, night watching, etc., may hasten its appearance.—*Medical Times.*

---

### TRICHLORACETIC ACID.

Dr. Stein, of Moscow, highly recommends trichloracetic acid in the treatment of nose and throat trouble. He says: "Weak solutions of trichloracetic acid used in the nose in simple atrophic rhinitis for a considerable time sometimes produce such a decided hypertrophy of the turbinated membranes that it may become necessary to cauterize them in order to secure free breathing. No other medicine of which the writer is cognizant produces such a remarkable effect, and thus this agent is particularly applicable in the treatment of ozena." In true ozena Dr. Stein applies stronger solutions than he did at first. He now uses a solution of one-half to ten per cent. in the treatment of ozena, and says that the odor is not so quickly nor so thoroughly abolished by any other medicinal agent as by the acid. In solutions of 1-to-500 to 1-to-2000 it will keep all suppurative processes in abeyance for a week. In dilute solution it is one of the best antiseptic remedies that Dr. Stein has ever employed, and it causes greater stimulation of atrophic membrane than any other application with which he is acquainted.—*The Pharmacologist.*

---

### CROUPOUS TONSILLITIS.

In the cases of adults the writer has in many instances aborted follicular tonsillitis by the following method: Each affected crypt was in turn washed out with peroxide of hydrogen, by means of a Blake's middle-ear canula screwed on to a hypodermic syringe. The curved tip of the canula employed is about one-half inch in length and capable of reaching to the bottom of the follicle. Only a drop or two of the peroxide is injected at one time, but the process is repeated until all of the exudate has disappeared. A fine Allen's probe with a few fibers of cotton wrapped about its end is then bent at an appropriate angle and after being dipped into a solution of nitrate of silver, one drachm to the ounce, is carried to the bottom of a follicle and the process repeated until each of the affected crypts have received the silver solution. The surface of the tonsil is then painted with the same solution. The treatment is followed immediately by a sense of relief and comfort and the difficulty in swallowing is in great measure alleviated. The process may be repeated two or three times a day and in successful cases brings about a cure at the end of the second or third day.—*Dr. Gleason, Atlantic Med. Weekly.*

---

### RECORD OF MEDICO-SURGICAL PRACTICE WITH AUXILIARY BLOOD-SUPPLY—"HEMATHERAPY"—(OR OTHERWISE) AT SOUND VIEW HOSPITAL, T. J. BIGGS, M.D., STAMFORD, CONNECTICUT.

*Catalogue Case No. 40. Tubercular Nephritis. Operation.*— T—— H——, Springdale, Conn.; male; English; age 12; admitted April 15, 1898. Three weeks before patient had been seized with a violent chill, followed by fever; and although the fever had been reduced under treatment, the temperature could not be gotten under 100.5. He had lost flesh rapidly; suffered great pain in the right kidney; passed large quantities of light urine, in which some blood was occasionally present. Microscopic examination of urine disclosed numerous tube casts and tubercle bacilli. Chemically, it showed a half of one per cent. albumen. I suggested operation, deeming it the only wise plan in view of the condition being so painful and progressing so rapidly. This was refused. I then put the patient on a teaspoonful of bovinine in milk every two hours, and half a drop of kreasote and a teaspoonful of sanmetto every three hours. Under this treatment he showed some improvement up to 22d; after which the pain and general symptoms of the previous condition returned. I again strongly advised operation, which was again refused. On the 28th, told the parents that if they would not consent to do as I said, absolutely in every detail, I would no longer assume responsibility in the case. On the 29th they consented to permit me to do as I saw fit. Consequently, after a day of preparatory treatment, was operated on, May 1st. An exploratory incision was made posteriorly, and the kidney was brought to the surface of the wound. It was found tremendously congested, and presenting the appearance of a well-defined case of renal tuberculosis. So thoroughly was the kidney involved, that on account of the child's weakened condition I deemed it unwise to allow it to remain, and therefore removed it. I found that the surrounding tissues gave no sign of tubercular deposit, but seemed to be in a thoroughly healthy condition. After cleansing the cavity with peroxide-on-bovinine and Thiersch irrigation, a glass drainage-tube was inserted, and the edges of the external wound were brought together around it. Bovinine was applied four times a day to the stump of the kidney, through the glass tube, until the 12th; when, being found in a healthy condition, the drainage-tube was removed, and the external edges of the wound were brought in apposition with one silver-wire suture and six silk sutures. On the 24th this wound was entirely healed, and on May 27, 1898, the patient was discharged cured.

Remark: The rapidity with which the condition in this case was healed is undoubtedly due to supplied blood, and a parallel to it I do not know.

*Catalogue Case No. 39. Chronic Salpingitis. Condemned Ovary Saved.*—Mrs. McC——, Stamford, Conn.; American; age 32; admitted April 11, 1898: Salpingitis of left ovary. Had been under the care of a leading physician, who advised her to have the ovary removed. This was absolutely refused, and I was called in consultation. I did not agree with my colleague that the removal was absolutely necessary. This pleased the patient so much that she decided to enter the hospital for treatment. Digital examination revealed a soggy mass posteriorly on the left side, the womb considerably retroverted, and severe endometritis. My theory was that absorption had taken place through the tube, and that if the endometritis were thoroughly cured the ovarian condition would subside, there being no positive evidence as yet of any pus. I therefore decided to put the patient on the following course of treatment: A teaspoonful of bovinine in old port wine every two hours, with a hot vaginal douche of plain sterilized water. This treatment was continued to the 27th, when the pain, which had been previously very severe, was entirely relieved. On the 28th, after etherizing the patient, I thoroughly curetted the womb, and after depuration with the bovinine-peroxide reaction, packed it with bi-sterilized gauze saturated with iodoform-bovinine. This was removed in forty-eight hours, the womb was again bovinine-peroxidized, and repacked with gauze saturated with bovinine pure. These depurations and packings were repeated until May 5th, when they were discontinued, and bovinine tampons were applied twice a day. The bovinine by mouth was increased to a wine-glassful in grape-juice every four hours. Patient now felt, as she expressed it, well and happy, aside from the weakness resulting from former sufferings. The bovinine tampons continued to be applied until the 20th, when the womb was found in a normal condition, there was no tenderness over the ovary, and the patient's general condition was better than it had been for years. A Thiersch douche was now employed at bedtime, up to the 28th. May 29, 1898, she was discharged cured, and delighted that her ovary had been saved.

*Dispensary Cases* (*T. J. B.*). *Caruncle of Urethra—Ulceration cxxv.*—Marie B——, New Canaan, Conn.; American; age 45; first seen May 3, 1898; Caruncle of urethra. History: Six months previously she had found it necessary to consult a physician, having suffered much distress during urination, followed by excruciating pain after the bladder was emptied. The physician told her that she had cancer of the meatus and first part of urethra, and advised immediate operation for removal of the same. Accordingly, a few days later, under etherization, a growth was partially removed. The patient reacted well from the immediate effects of the operation, but now experienced greater pain than ever, which was continuous. She consulted another physician, who put her through a course of treatment, but in spite of this she steadily grew worse.

On May 3, 1898, she came under my care. Examination revealed a large-sized caruncle, involving the entire circumference of the meatus and from a half to three-fourths of the anterior portion of the urethra. Laterally, on the right side, was a very hard and painful cicatrix, resulting from the former operation. I advised operation, and the patient consented. But her condition being one of pronounced neurasthenia and general debility, I deemed it wise to put her on a few days of preparatory treatment before operating. After regulating the secretions I ordered given her a teaspoonful of bovinine in milk and grape-juice alternately, every two hours. May 6th, the patient's condition being favorable, she was chloroformed and the growth and scar were both carefully dissected out. The urethra was now packed gently with a strip of bi-sterilized gauze packed with bovinine pure. Incidentally it may be mentioned that the urethra was thoroughly dilated, so that incontinence of urine might result for a few days, to prevent contraction of the urethra down on the denuded surface of the wound. At the end of twelve hours the packing was removed, and urethra and bladder were washed out. A glass tube, prepared with many perforations all over its circumference, was now inserted, and held in place by an improvised harness; the object being to not only thoroughly drain the bladder, thereby giving the muscles rest and keeping the denuded surfaces apart, but also at the same time provide for feeding the denuded surfaces by dropping in and diffusing bovinine, which was done hourly the first day, and every two hours the second day. This tube was allowed to remain in until the 10th, when it was removed, and the urethra was found to be in a healthy granulating condition. Bovinine pure was now injected with a glass syringe every two hours, and urine drawn three times a day, until the 16th, when the surfaces were almost entirely healed, the normal power of the urethra was restored, and urine was vóided without any pain; the only part not healed being the site of the old scar made by the former operation. May 21st, this also being healed, the patient was discharged absolutely cured.

